# The MRPS18-2 protein levels correlate with prostate tumor progression and it induces CXCR4-dependent migration of cancer cells

**DOI:** 10.1038/s41598-018-20765-8

**Published:** 2018-02-02

**Authors:** Muhammad Mushtaq, Lasse Jensen, Sabina Davidsson, Oleksandr V. Grygoruk, Ove Andrén, Vladimir Kashuba, Elena Kashuba

**Affiliations:** 1 Department of Microbiology, Tumor and Cell Biology (MTC), Karolinska Institutet, Stockholm, 17177 Sweden; 2 Linköping University, Linköping, 581 83 Sweden; 3 Department of Urology, Faculty of Medicine and Health, Örebro University, Örebro, 70182 Sweden; 4 Clinic Boris, 12AM. Bazhana ave, Kyiv, 02140 Ukraine; 5 Institute of Molecular Biology and Genetics, NASU, 150 Zabolotnog str, Kyiv, 03143 Ukraine; 6 R.E. Kavetsky Institute of Experimental Pathology, Oncology and Radiobiology, NASU, 45 Vasylkivska str, Kyiv, 03022 Ukraine

## Abstract

We have earlier found abnormal expression of the mitochondrial ribosomal protein S18-2 (MRPS18-2, S18-2) in endometrial cancer, compared to the expression in hyperplasia and in normal endometrium. Here we report that expression of S18-2 was increased with disease progression in clinical specimens of prostate cancer (PCa). The level of induction of epithelial to mesenchymal cell transition (EMT) correlated with the expression level of S18-2 in PCa cell lines. Moreover, cells acquired increased ability of migration upon S18-2 overexpression, as was evaluated in zebrafish embryo model and in trans-well assay. We found that this is due to increased CXCR4 cell surface expression. Neutralizing CXCR4 protein or abrogating S18-2 expression in cells significantly reduced their migratory ability directed toward CXCL12. The mRNA expression of *TWIST*2, encoding one of transcription factors that induce EMT upon CXCR4 increase, positively correlated with the S18-2 protein level. Together, these data suggest that the S18-2 protein induces EMT through the TWIST2/E-cadherin signalling and, consequently, CXCR4-mediated migration of PCa cells.

## Introduction

Prostate cancer (PCa) is the most frequently diagnosed cancer in men. According to the Swedish national board of health and welfare (Socialstyrelsen) database, 10440 men were diagnosed with PCa and 2357 PCa related deaths were recorded in Sweden in 2015 (Available at: http://www.socialstyrelsen.se/statistik/statistikefteramne/cancer).

Prostate cancer is the malignancy of prostate epithelial cells. Most of the PCa–related deaths are due to the advanced stage of the disease that results from any combination of lymphatic, hematogenous, or contiguous local spread. The mechanisms underlying PCa metastasis are incompletely understood^1^. Therefore, identification of potential prognostic biomarkers for PCa are relevant to better therapeutic intervention in metastatic PCa.

Our previous finding shows that the expression level of S18-2 (NP_054765) and the free E2F1 (NP_005216) protein increased with the advancement of endometrial cancer (EC)^2^. The S18-2 signal was not observed in normal tissues; very weak signal was detected in hyperplasia. In contrast, tumor cells demonstrated strong cytoplasmic S18-2 staining. In low differentiated endometrial adenocarcinoma a small proportion (3-5%) of cancerous cells showed nuclear signal of S18-2. Noteworthy, the high E2F1 expression correlated with highly expressed S18-2 in analyzed tumors^2^.

The rationale behind such expression pattern is based on the fact that S18-2 binds to retinoblastoma associated protein (RB, NP_000312), thus freeing from a complex with RB the E2F1 transcription factor^3,4^. The ectopic expression of S18-2 induced epithelial to mesenchymal transition (EMT) in EC derived cell lines. Simultaneously, epithelial markers E-cadherin (NP_004351), pan-keratin and β-catenin (NP_001895) were reduced upon S18-2 overexpression in EC cells while vimentin was induced^2^.

The RB-S18-2 interaction results in advancement of S-phase of the cell cycle; consequently, it leads to immortalization of primary cells^3,4^. Furthermore, S18-2 induces chromosomal instability and, eventually, transformation of primary cells, accompanied with a de-differentiated phenotype^5,6^. In colon adenocarcinoma, a specific polymorphism of S18-2 was frequently found^7^.

Noteworthy, more than 2 000 genes were differentially expressed in cells immortalized by overexpression of S18-2 compared to control cells. For example, elevated expression of SOX2 (NP_003097), OCT4 (NP_976034), Nanog (NP_001284627) and CXCR4 (NP_001008540) was observed^6^.

Here we report that S18-2 protein levels are elevated in low differentiated prostate tumors. Moreover, in PCa tissues a strong CXCR4 signal was observed in cells expressing high levels of S18-2. Using sub-lines of PC3 cells (derived from PCa) overexpressing S18-2, we show enhanced expression of CXCR4 on cell surface. This, in turn, leads to increased migration of cancerous cells, both *in vitro* (in a CXCL12 (NP_954637) directed trans-well assay) and *in vivo* (in a zebrafish model). The increased migration is due to EMT, induced by S18-2 via repression of E-cadherin by *TWIST2*, explaining in a part the aggressiveness of cancer.

## Results

### The expression of the S18-2 protein is increased in tumors compared to in hyperplasia and in normal tissues

**Figure 1:**
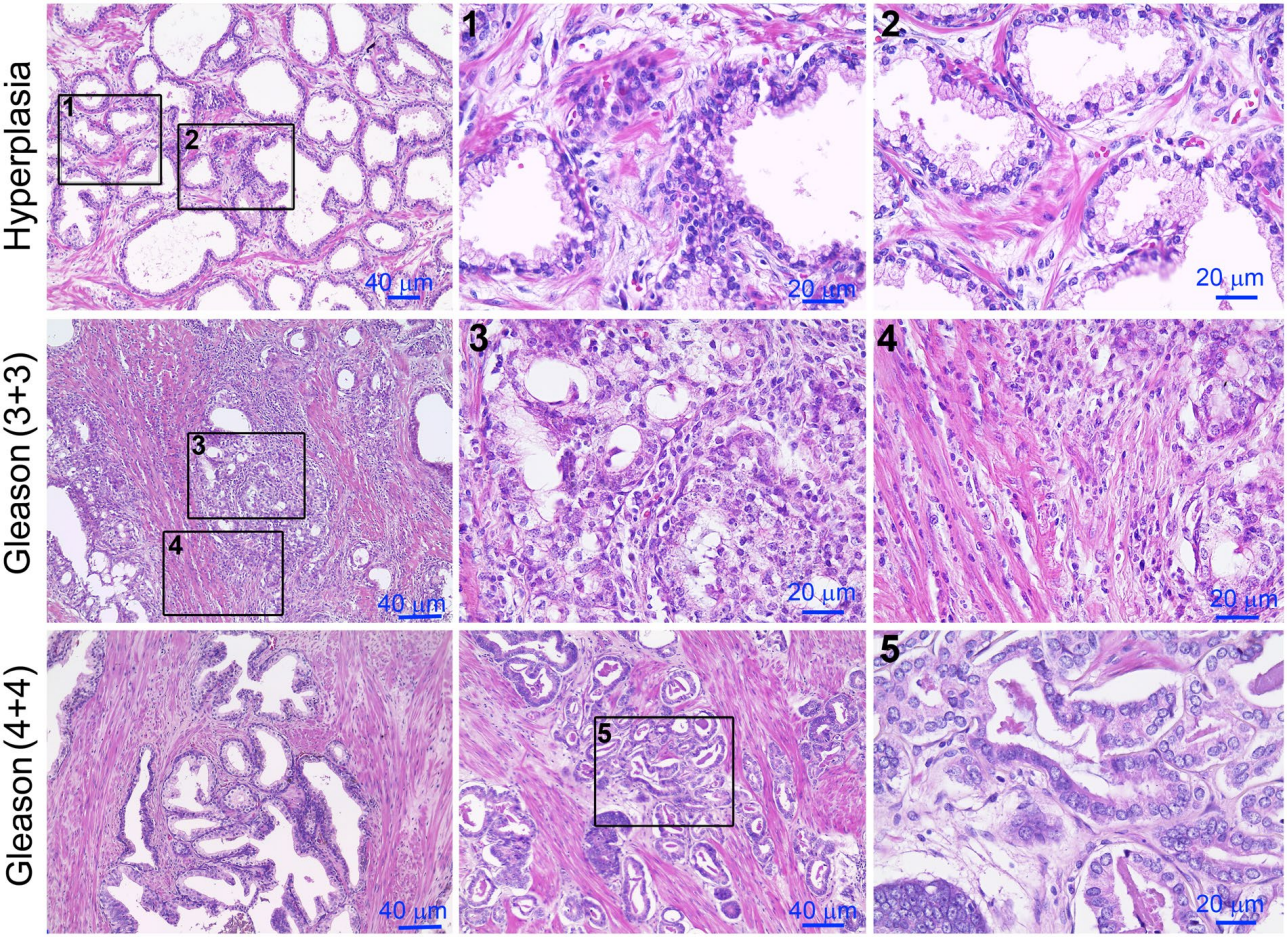
Hematoxylin-eosin (HE) staining of whole prostate tissues. The top row – hyperplasia; see squares 1 and 2 for larger magnification (the top row). The middle and bottom rows - PCa of Gleason 6 and 8, respectively.

The expression level of S18-2 was analyzed in PCa of different grades and normal prostate tissues. For this purpose, the whole prostate tissues from 23 patients who underwent radical prostatectomy were used. In total, 12 out of 23 patients were diagnosed with PCa (Gleason score ≥6). Prostate tissues of the remaining 11 patients with hyperplasia and/or inflammation in some lesions were used as controls (see supplementary Table S1). The PCa tissues were analyzed by two experienced pathologists independently, to identify the hyperplastic and neoplastic lesions in the samples. Representative images of prostate tissues are shown on Fig. 1.

**Figure 2:**
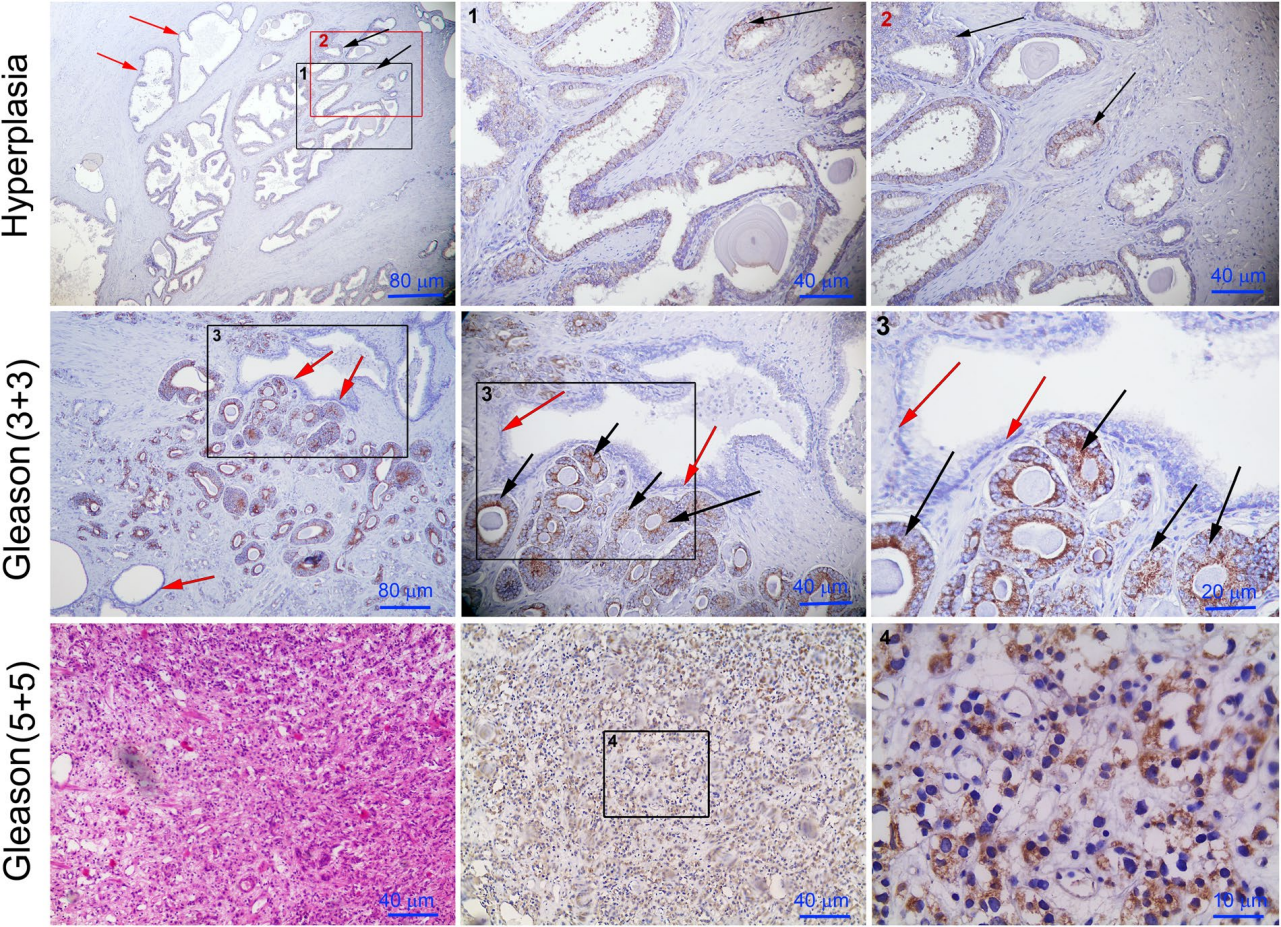
Expression of the S18-2 protein in prostate tissues. Whole prostate tissue (sections) was stained with the S18-2 specific antibody. The top row – hyperplasia. The S18-2 signal was almost absent in the normal glands (red arrows); see squares 1 and 2 for larger magnification. Notice the S18-2 signal in a proportion of the gland cells (black arrows). The middle row – PCa of Gleason 6, the bottom row – PCa of Gleason 10. Notice the large cells with the prominent nucleoli (the middle row, black arrows). See the squares 3 and 4 for larger magnification. The S18-2 is expressed in every cell of the low differentiated PCa (the bottom row). See the left panel in the bottom row for HE staining.

We observed that the S18-2 signal was relatively high in cancerous sections (Fig. 2). In contrast, a comparatively weak S18-2 signal was detected in some sections of the PCa hyperplasias; however, almost all hyperplastic lesions were negative (Fig. 2, the top row). Of note, the strong S18-2 signal was observed throughout the lesions in the aggressive adenocarcinomas (Fig. 2, the middle and bottom rows). Interestingly, the cells in these lesions have prominent nucleoli (Fig. 2, the middle and bottom rows, indicated by black arrows). The lumens of S18-2 expressing glands were smaller; cells were round and uniform in size (Fig. 2). In benign glands, characterized by large and branched lumen, cells were negative for S18-2 expression (Fig. 2, the top and bottom rows). Moreover, the S18-2 expressing lesions lacked the basal cell layer, indicating the neoplastic gland, while the neighboring benign gland possessed the layer of basal cells (Fig. 2, the bottom row). Of note, cells in such lesions did not express S18-2 (Fig. 2, the bottom row, indicated by red arrows).

To confirm our experimental data, the mRNA expression pattern of *S18-2* was analyzed, using a publically available database Oncomine. This data base contains published data that has been collected, standardized, annotated and analyzed by Compendia Bioscience (www.oncomine.com, November 2017, Thermo Fisher Scientific, Ann-Arbor, MI, USA). The data showed that S18-2 expression is tightly correlated with progression of disease, as the expression of S18-2 was higher in prostate adenocarcinomas and metastatic samples compared to normal prostate tissues. The upregulated expression of S18-2 was also correlated with the increase of Gleason score (Supplementary Figure S1).

### The degree of EMT induction in PCa cells correlates with the expression level of S18-2

**Figure 3:**
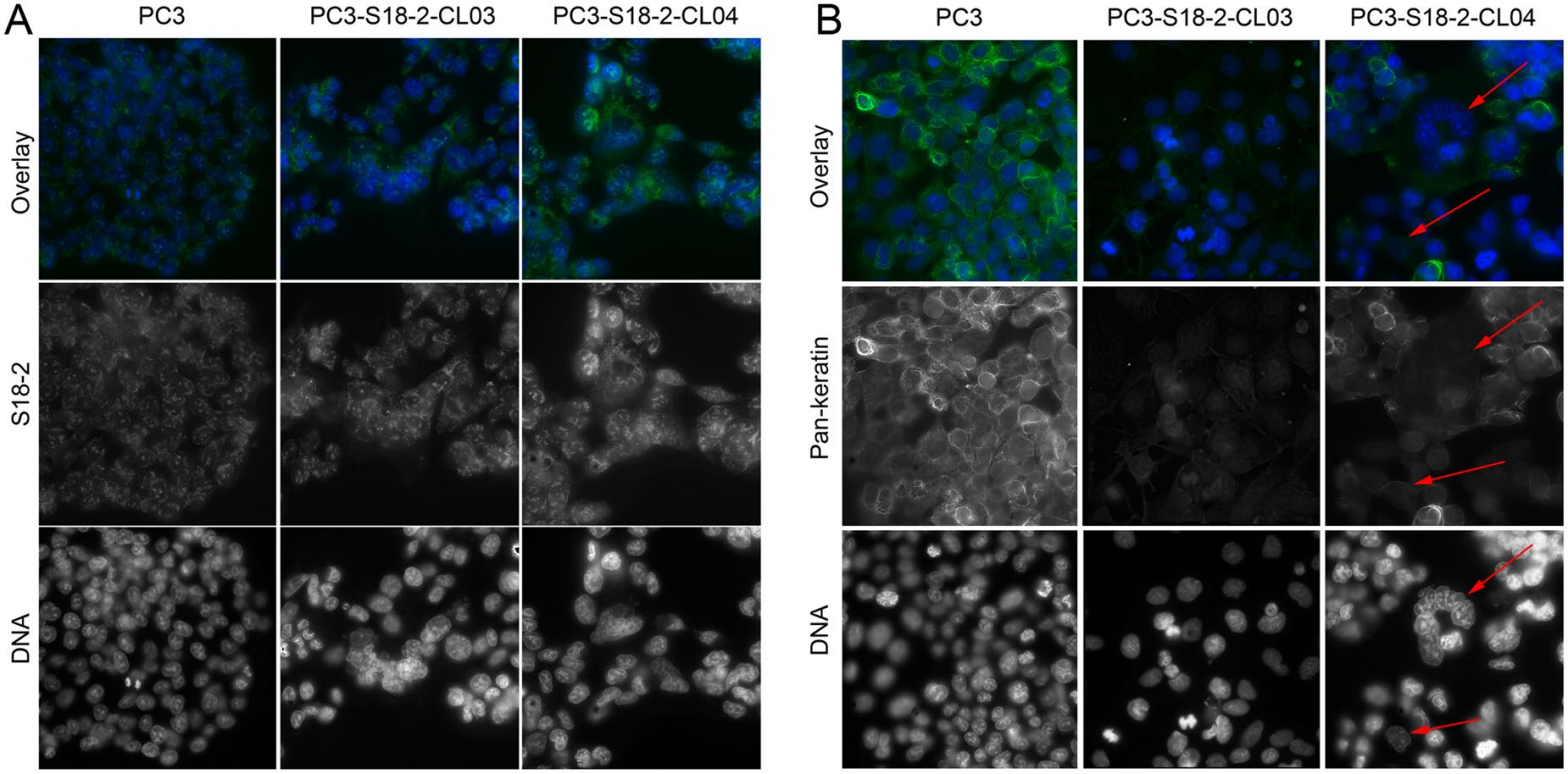
Immunofluorescent staining of the different PC3 cells sub-lines. Cells were stained with specific antibodies against the S18-2 protein (**A**) and pan-keratin (**B**). Notice the strong S18-2 signal (green, when overlaid; white, when alone) in all cells. The strongest S18-2 signal was detected in PC3-S18-2-CL04 cells (the left panel, the right column). At the same time, the pan-keratin signal (green, when overlaid; white, when alone) was weak in sub-lines. Notice the low expression of pan-keratin in PC3-S18-2-CL04 cells, especially in multinucleated cell in the middle (indicated with red arrows).

**Figure 4:**
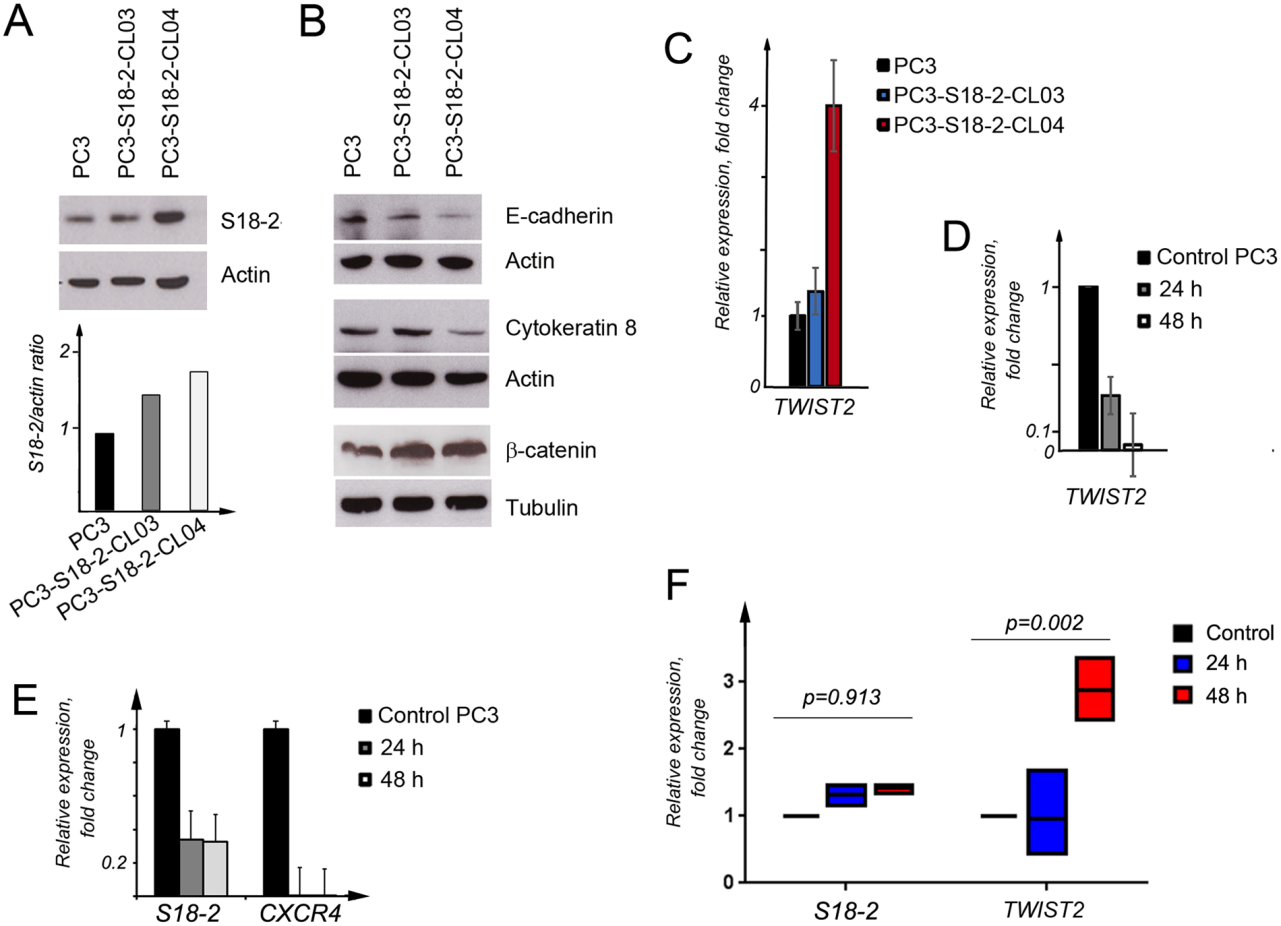
The expression level of EMT induction markers. (**A**) Western blot analysis showing the expression level of S18-2 in PC3, PC3-S18-2-CL03 and PC3-S18-2-CL04. The graph shows the intensity of S18-2 bands, normalized to the intensity of corresponding actin bands. (**B**) Western blotting showed that E-cadherin and cytokeratin 8 was decreased at the protein levels in PC3-S18-2-CL04 compared with PC3 cells. The expression of β-catenin was not changed among the three cell lines. Actin and Tubulin were used as loading controls, respectively. Scans of all gels are presented in Supplementary Figure S2. (**C**) The q-PCR analysis of *TWIST2*. The *TWIST2* was expressed at significantly higher levels in PC3-S18-2-CL04 than in the control cells. (**D**) The *TWIST2* mRNA expression after 24 and 48 h of S18-2 downregulation. The *TWIST2* gene was downregulated significantly upon *S18-2* knocking down by siRNA in PC3 cells. (**E**) Expression level of *S18-2* and *CXCR4* in PC3 cells after 24 and 48 h of the treatment of PC3 with *S18-2* specific siRNA. As expected, *S18-2* was reduced with transfection of *S18-2* specific siRNA compared to control siRNA treated cells. CXCR4 was also significantly reduced in cells transfected with S18-2 specific siRNA compared to control siRNA treated PC3 cells. (**F**) the mRNA expression level of *S18-2* and *TWIST2* after activation of CXCR4 by CXCL12 treatment. Cells were treated for 24 and 48 h. The *TWIST2* gene was induced after 48 h. The *S18-2* expression was not affected by CXCL12 treatment. All the experiments were repeated at least three times. Medians of three q-PCR reactions were analyzed, using the GraphPad Prism software. Unpaired t test was applied and two tailed p values for each experiment (controls −3, 24 h −3, 48 h −3 values) were determined.

Taking into consideration the pattern of S18-2 expression in prostate tumors and the fact of induction of EMT in EC cells^2^, we generated PC3 sub-lines overexpressing S18-2 and mock-transfected cells for further studies. These sublines, PC3-S18-2-CL03 and PC3-S18-2-CL04, expressed the S18-2 protein at different levels, as was shown by immunostaining (Fig. 3, the left panel, the top and middle rows) and western blotting (Fig. 4A) with a specific antibody. Noteworthy, levels of EMT markers correlated with the intensity of the S18-2 protein signal. Intensity of the pan-keratin signal was lower in clones, compared with the parental PC3 cell line (Fig. 3B). The staining pattern of pan-keratin is heterogeneous though – some cells in clone showed the higher signal intensity, some (indicated by red arrows on Fig. 3B, the right panel) showed almost no signal. Overall, pan-keratin was lower in clones, compared with PC3 cells. Moreover, levels of cytokeratin 8 (NP_001243211), and E-cadherin were reduced in PC3-S18-2-CL04, compared with PC3, as is shown by western blotting (Fig. 4B). Together, these data suggest that EMT was induced in PC3-S18-2-CL04 to a higher degree compared to PC3 and PC3-S18-2-CL03.

In order to answer the question what transcription factor(s) involved in EMT induction upon S18-2 overexpression, mRNA expression of the genes, encoding a set of EMT related transcription factors, was assessed, using real time quantitative PCR (q-PCR). The q-PCR analysis was performed for PC3-S18-2-CL03 and PC3-S18-2-CL04 cells, and also upon downregulation of *S18-2* in PC3, using siRNA. No significant difference was observed for any of the genes analyzed (*KLF8* (NM_007250), *SNAIL* (NM_005985), and *ZEB1* (NM_030751)) when S18-2 protein was overexpressed, except for the *TWIST2* (NM_057179) gene (Supplementary Figure S3A). In PC3-S18-2-CL04 cells expressing S18-2 at very high levels, expression of *TWIST2* was 4-fold higher, than in PC3 (Fig. 4C). Importantly, when the parental PC3 cells was treated with pool of siRNA, specific for *S18-2*, expression of *TWIST2* was reduced significantly (3-fold) after 24 h of the treatment, and was barely detectable after 48 h (Fig. 4D and Supplementary Figure S3B). Meanwhile, changes in the expression of three other genes were not observed. We performed three more transfections of PC3 cells, using a mixture of siRNA, specific for *S18-2*, and observed similar changes in *TWIST2* and *S18-2* expression, after 24 h (Supplementary Figure S3C).

### CXCR4 expression correlates with S18-2 expression, while CXCR4 activation led to increase in TWIST2 levels

**Figure 5:**
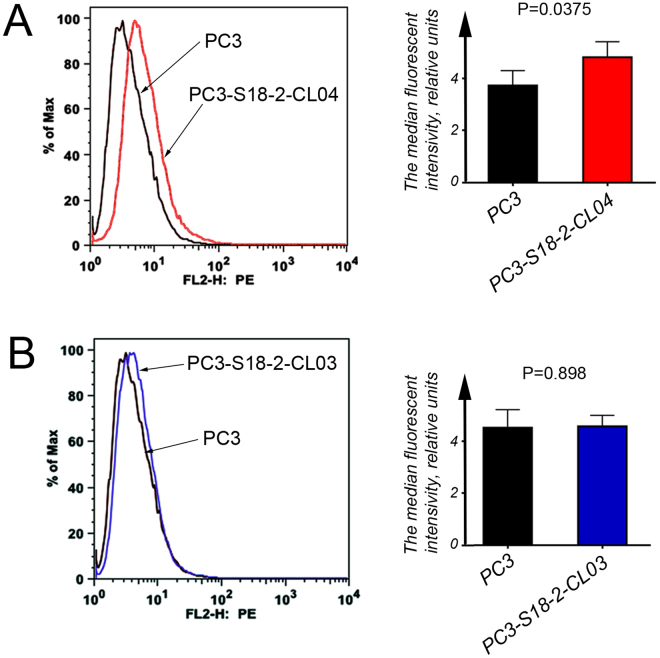
The CXCR4 cell surface expression. (**A**) Flow cytometric histogram of PC3 versus PC3-S18-2-CL04 (the left panel). Notice the increase in CXCR4 expression in PC3-S18-2-CL04 cells, as there is shift in the histogram peak (red color). The median fluorescence intensities were analyzed and statistical analysis indicated that PC3-S18-2-CL04 cells (the right panel) express CXCR4 at a significantly higher level, than PC3. (**B**) As (**A**), but PC3-S18-2-CL03 cells were compared with PC3. The expression of CXCR4 was similar in PC3 and PC3-S18-2-CL03 (in blue color). All experiments were repeated 4 times. Graphs, presented in the right panels, were generated, using the statistical analysis with GraphPrism6 software.

It was shown previously that PC3 cells express the chemokine receptor CXCR4^8^. Hence, we decided to assess surface expression of the CXCR4 protein, using a flow-cytometric analysis (FACS) and specific antibodies. Importantly, overexpression of S18-2 led to a statistically significant increase in CXCR4 expression (p = 0.038) in PC3-S18-2-CL04 cells (Fig. 5A). The CXCR4 protein levels were not changed in PC3-S18-2-CL03 cells, compared with parental PC3 line (p = 0.9) (Fig. 5B). Importantly, when *S18-2* was downregulated upon transfection of cells with a cocktail of specific siRNA, *CXCR4* was also reduced at the mRNA level (Fig. 4E). Three independent S18-2 specific siRNA treatment experiments were repeated and *CXCR4* expression was analyzed after 24 h of transfection. The data is presented in Supplementary Figure S3D: *CXCR4* mRNA level was significantly reduced after downregulation of *S18-2* in the same manner as given on Fig. 4E.

Furthermore, we asked a question whether activation of CXCR4 by CXCL12 will result in changes of *TWIST2* levels, or not. To do so, the serum starved PC3 cells were treated with CXCL12. The *TWIST2* expression was detected at 24 and 48 h after beginning of the treatment with CXCL12. After 48 h a dramatic increase in the *TWIST2* expression was detected (Fig. 4F and Supplementary Figure S3E). Importantly, the CXCL12 treatment did not influence the *S18-2* expression (Fig. 4F and Supplementary Figure S3F).

### Cells expressing high levels of S18-2 showed higher CXCL12 directed trans-well migration

**Figure 6:**
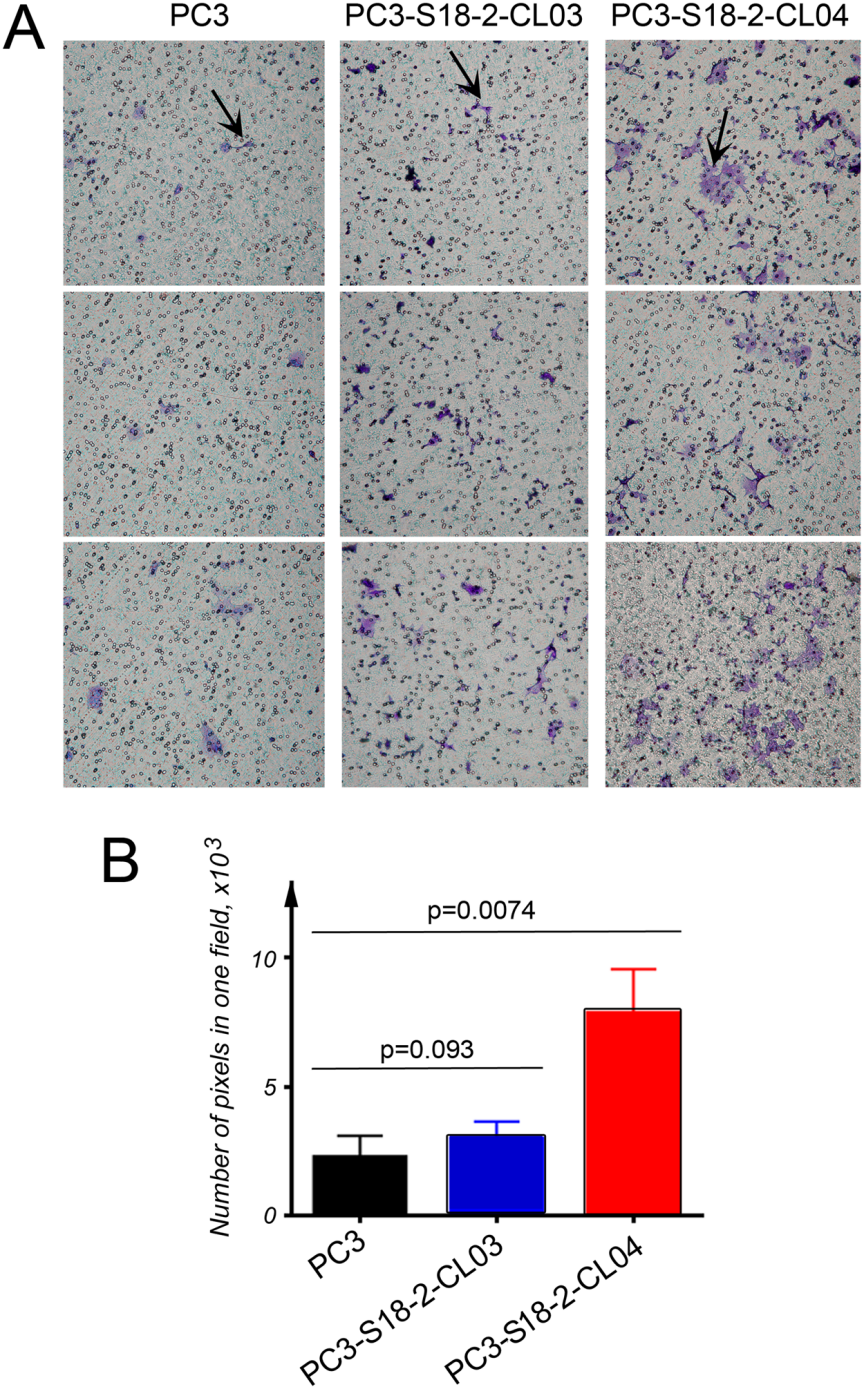
CXCR4-mediated *in vitro* migration. The cells were cultured in trans-well plates and their ability to migrate towards chemotactic CXCL12 was assessed. (**A**) Cells were stained with crystal violet and the migration of PC3 (the left panel), PC3-S18-2-CL03 (the middle panel) and PC3-S18-2-CL04 (the right panel) was assessed based on the number of cells that crossed the membrane. Representative images show migrated cells that are indicated with arrows. (**B**) The intensity of crystal violet signal was quantified as a measure of the cells. We performed these experiments in triplicates and in two independent sets. The statistical analysis, using the GraphPrism 6 software showed the significant increase (p < 0.05) in motility of PC3-S18-2-CL04 cells, compared with PC3.

The next step was to analyze the activation of the CXCL12-CXCR4 axis, resulting in cell migration. In order to monitor the CXCL12 directed migration *in vitro*, the trans-well assay was applied. Following overnight starvation of the cells, the next day cells were treated with CXCL12. Importantly, the trend in cell migration in response to CXCL12 followed the earlier observed dependences, i.e. the EMT markers at the protein and mRNA levels and *CXCR4* expression positively correlated with the increased amounts of the S18-2 protein. Significantly higher number of the migrated cells was detected in PC3-S18-2-CL04, compared with PC3-S18-2-CL03 (p = 0.093) and PC3 (p = 0.007) (Fig. 6).

### The S18-2 overexpressing cells showed the higher CXCR4-dependent migration ability in a zebrafish model

**Figure 7:**
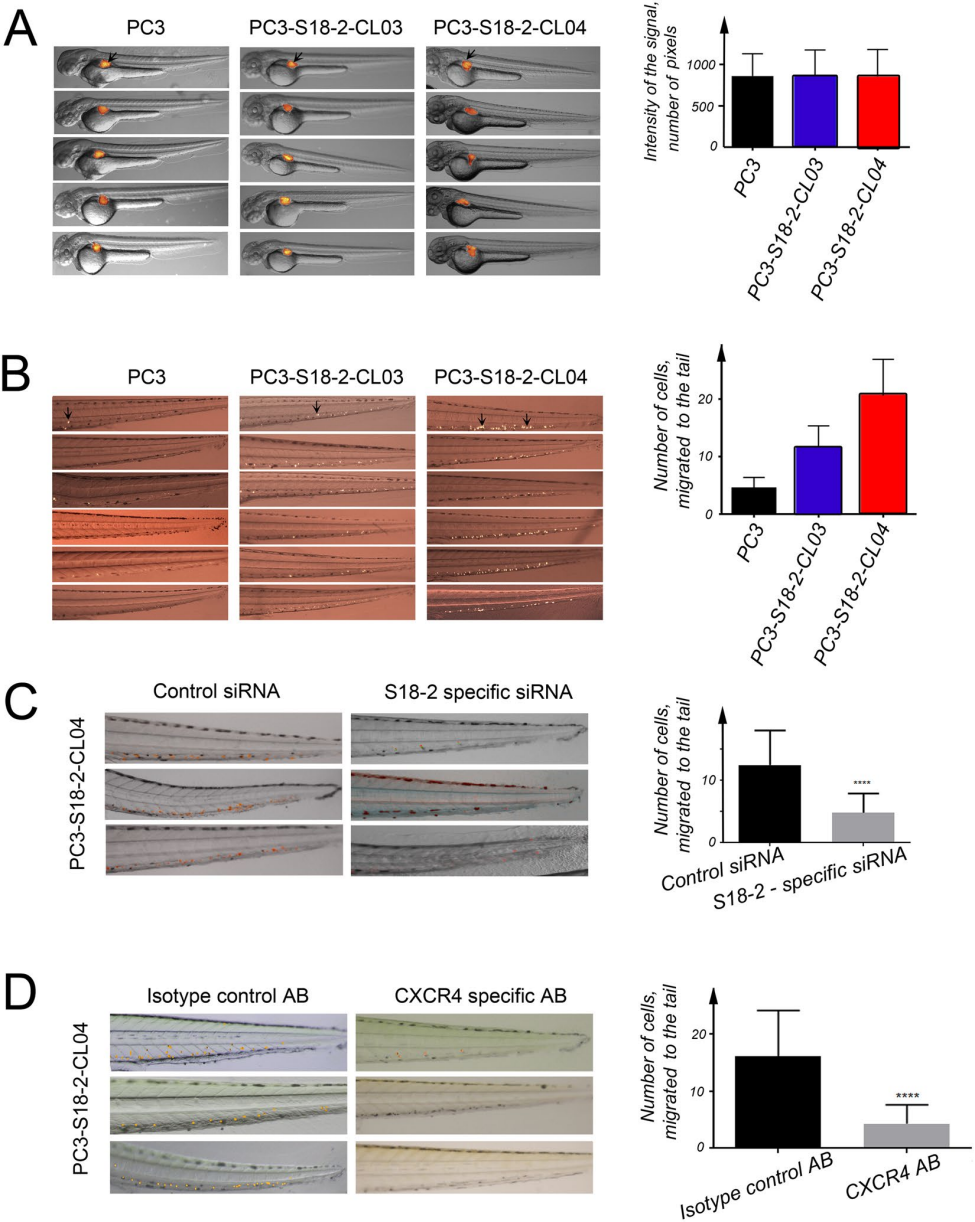
Study on motility of cells in a zebrafish model. (**A**) The number of DII labelled cell injected to perivitelline cavity of 48 hpf zebrafish embryos were similar for PC3 (the left panel), PC3-S18-2-CL03 (the middle panel) and PC3-S18-2-CL04 (the right panel) cells. (**B**) The number of cells migrated to the tail of 120 hpf zebrafish embryos (after 3 days of injection). The arrows indicate the cells migrated to the tail. Notice the larger number of migrated PC3-S18-2-CL04 cells, in comparison with PC3-S18-2-CL03 and parental PC3 cells. Statistical analysis was performed, using GraphPrism 6 software. (**C**) Migration of PC3-S18-2-CL04 to tail of zebrafish after down regulation of S18-2 using siRNA (the right panel). Embryos were also injected after treatment with control siRNA (the right panel). The graph shows significant difference after down regulation of S18-2. (**D**) After DII labelling cells were incubated overnight with α-CXCR4 (the right panel) or isotype control antibody (the left panel). Cells were injected into embryos along with CXCL12. Three days after injection cells were counted in both the groups. The graphical representation shows significant difference of migrated cells among the two groups. At least 20 embryos were injected every time, and experiments were performed minimum 3 times.

Due to the fact, that S18-2 protein induces EMT in PC3 cells, the migration ability (motility) of the parental and cells of sub-lines overexpressing S18-2, was monitored. A zebrafish embryo model was used. The DII labeled PC3, PC3-S18-2-CL03 and PC3-S18-2-CL04 cells were injected in the perivitelline space of zebrafish embryos at 48 hours post fertilization (hpf). The number of cells injected into embryos was similar for each cell type (Fig. 7A); outlier injected embryos were removed.

Importantly, we found that the number of PC3-S18-2-CL04 migrated cells was significantly higher compared with PC3-S18-2-CL03 and parental PC3 cells. The number of PC3-S18-2-CL03 migrated cells was between the values for PC3-S18-2-CL04 and the PC3 cells (Fig. 7B).

Hence, EMT induction upon S18-2 overexpression resulted in enhanced motility of cancerous cells. This effect is dose-dependent, i.e. the highest mobility was demonstrated by PC3-S18-2-CL04 cells where the highest expression of S18-2 was detected (see Figs 3 and 4A). Importantly, when S18-2 was downregulated by siRNA, the motility of the PC3-S18-2-CL04 cells was dramatically decreased (Fig. 7C).

We observed significant diminishing of cell migration ability when CXCR4 was blocked in PC3-S18-2-CL04 cells by a specific anti-CXCR4 antibody. This also caused reduction in the CXCL12 directed migration of cells, compared with the same cells incubated with an isotype control antibody in the zebrafish embryo model (Fig. 7D).

### Cells overexpressing S18-2 gave rise to smaller tumors in SCID mice

**Figure 8:**
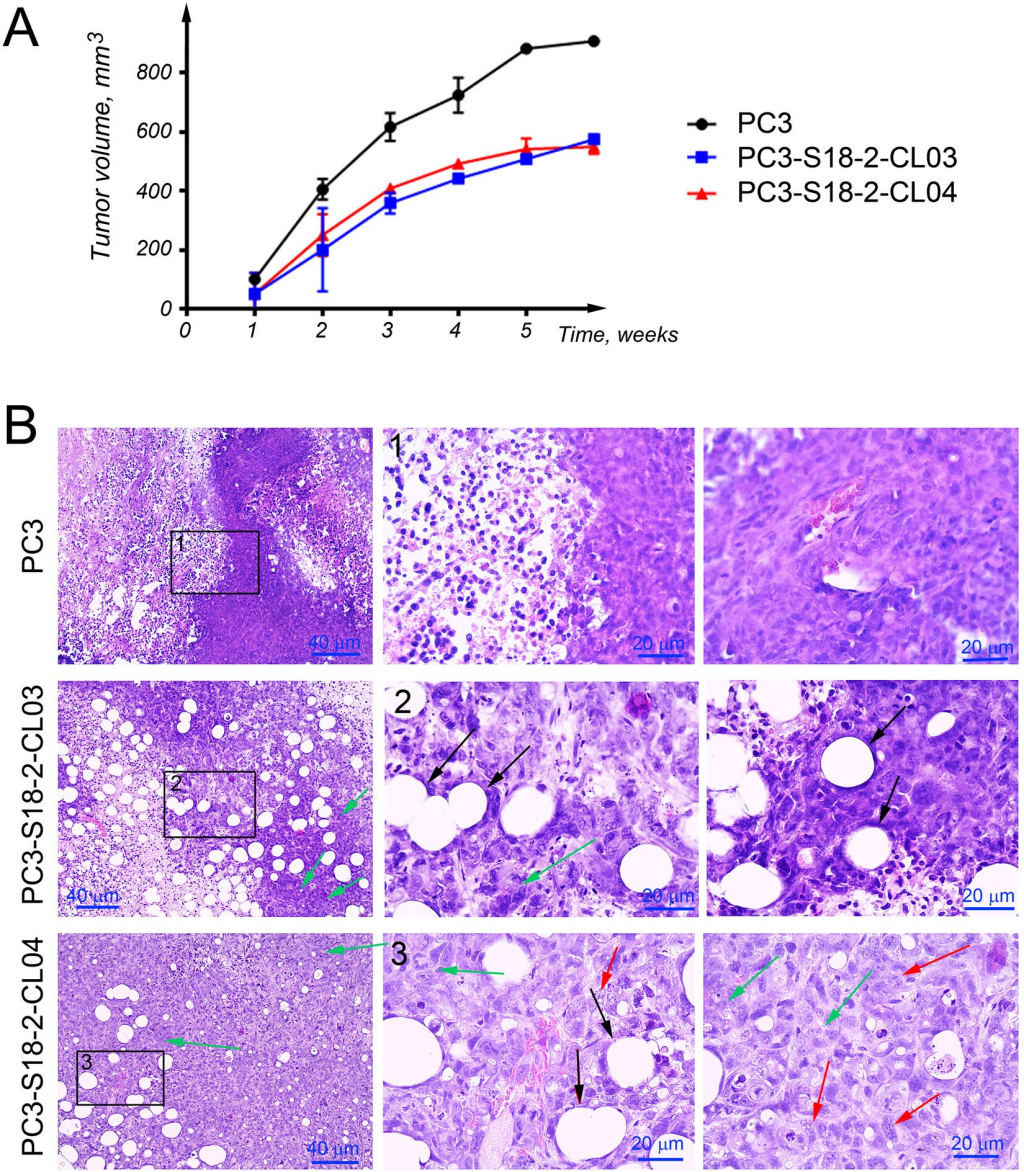
Tumorigenicity of the cells, overexpressing S18-2 in comparison with the control cells. PC3, PC3-S18-2-CL03 and PC3S18-2-CL04 cells were injected (2 × 10^6^) subcutaneously into SCID mice. (**A**) PC3-S18-2-CL03 and PC3-S18-2-CL04 gave smaller tumors compared to the control cells. (**B**) HE staining of tumors sections showed the formation of the dense tumors by PC3 cells (the top row). S18-2 overexpressing cells produced heterogeneous tumors, infiltrated by lymphocytes (the middle and the bottom rows). Many adipocytes were seen (black arrows). These cells produced multiple foci (green arrows). Also, the multinucleated cells were detected (red arrows).

To analyze the tumorigenicity of the parental PC3 line and sub-lines overexpressing the S18-2 protein, cells were introduced subcutaneously into SCID mice. Every mouse was injected into two different locations with 2 × 10^6^ cells. For each cell lines two mice were used (4 injections altogether) in order to obtain statistically significant observations. Surprisingly, PC3 cells formed larger tumors than both PC3-S18-2-CL03 and PC3-S18-2-CL04 cells. The PC3 cells had developed larger tumors more rapidly, compared with the tumors from PC3-S18-2-CL03 and PC3-S18-2-CL04 cells (Fig. 8A). Noteworthy, the morphology of the tumors was quite different. The PC3 cells produced dense tumor tissues, with some necrotic areas and infiltration of lymphocytes (Fig. 8B, the top row). In the tumors formed by PC3-S18-2 cells plenty of the infiltrated lymphocytes and many adipose cells were observed (black arrows, Fig. 8B). In addition, these tumors contained many foci and buds (green arrows, Fig. 8B). Moreover, tumor cells were highly aneuploid and many were multinucleated (red arrows, Fig. 8B). This, in turn, is a strong indication of metastatic tumor with more migratory ability^9,10^.

### CXCR4 expression pattern was similar to that of S18-2 in human tumors

**Figure 9:**
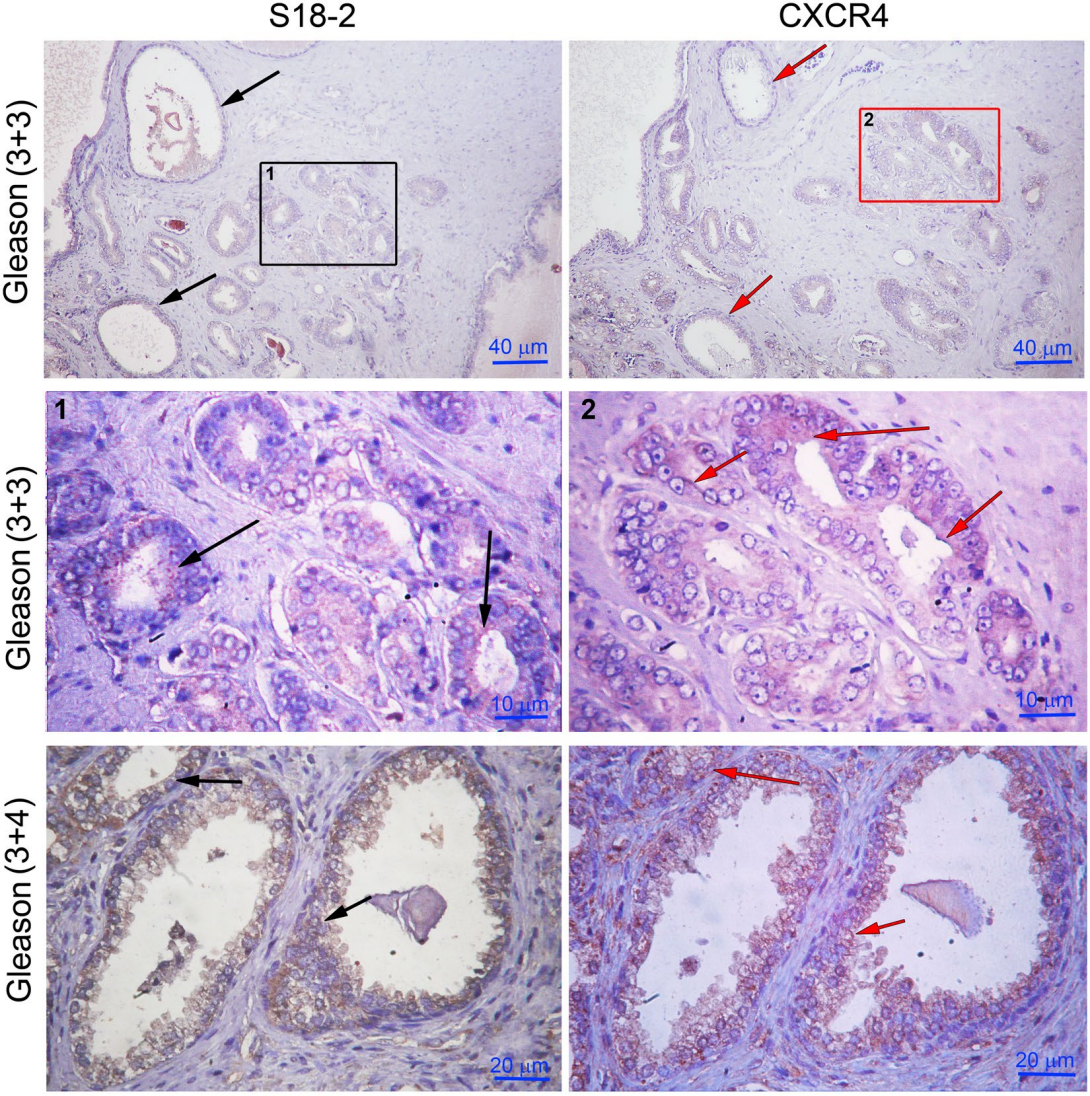
Expression pattern of CXCR4 and S18-2 in serial sections of PCa. The expression of S18-(left panel) and CXCR4 (right panel). Both proteins were expressed in common lesion (the lower and middle rows). In those tissues the cells have prominent nuclei as denoted by arrows in the middle row. Noteworthy, the CXCR4 expression was not observed in glands which are negative also for S18-2 as shown by red and black arrows in upper rows.

To investigate a possible relationship between S18-2 and CXCR4 expression, serial sections of PCa specimens were stained with the corresponding antibodies. Noteworthy, in the majority of neoplastic glands the expression pattern of S18-2 and CXCR4 was quite similar (Fig. 9, the middle and bottom rows, S18-2 is indicated by black arrows, CXCR4 by red arrows). However, few lesions were positive only for CXCR4. Importantly, in benign lesions which were negative for CXCR4, no S18-2 signal was detected (Fig. 9, the top row). This is in agreement with the data reported earlier, that expression of CXCR4 increases with progression of PCa^11^.

## Discussion

As mentioned above, PCa is the most commonly diagnosed cancer and the second leading cause of cancer-related deaths in men^12^. It is known that the early genetic and epigenetic events in the transition from normal prostate to PCa involves the loss of *FOXP3* (NM_014009), downregulation of *glutathione-S-transferase P1* (NM_000852) and *NKX3-1* (NM_006167) expression, increase in lipid metabolism and overexpression of *SPINK1* (NM_003122) and also genes, encoding the ETS Family of transcription factors, due to chromosomal translocations^13^.

It was also reported that the conversion of prostate tumor to high-grade carcinoma and metastasis requires further changes, namely loss of *PTEN* (NM_000314), *EPHB2* (NM_017449) and microRNA-101, *c-MYC* amplification (NM_002467) as well as overexpression of Hepsin (NP_002142), PIM1 (NP_001230115) and EZH2 (NP_004447)^13^.

As we discussed above, PCa is an epithelial neoplasm and the stroma cells of the prostate play an important role in the tumor progression. Particularly, the metastasis of tumors is known to be induced by stromal cells of the cancer tissue by enhancing EMT. The cross talk between tumor and stromal cells is partially mediated through chemokines.

CXCL12-CXCR4 is one of the most appreciated chemokine signaling axis that plays a critical role in progression of various solid tumors^14^. CXCL12 (also called stromal cells derived factor 1, SDF1) and its receptor, CXCR4 (a G protein-coupled transmembrane receptor) are ubiquitously expressed^15^. CXCL12 belongs to a chemokine family of small peptides (8 to 12 kDa in size) that are involved in the control of cell activation, differentiation, and migration^16,17^. It was shown, analyzing more than 600 PCa specimens that the CXCR4 protein expression increased with tumor progression. Moreover, the levels of CXCL12 were higher in metastatic lesions, than in the primary tumors^11^.

It was reported earlier that the CXCL12–CXCR4 axis modulates PCa cell migration, metalloproteinase expression and invasion^8^. Transcription factor SLUG (NP_003059) promotes PCa cell migration and invasion through CXCR4-CXCL12 axis^18^. Also, the CXCL12-CXCR4 axis promotes intraosseous tumor growth by transactivation of growth factor receptors like HER2 (NP_001005862) and EGFR (NP_001333826) in lipid rafts of PCa cells^19^. The small GTP protein G_αi2_ (NP_002061) is required for Src (NP_005408) and HER2 phosphorylation in lipid raft membrane microdomains^20^. In PCa preclinical models was demonstrated that pharmacological inhibition of CXCR4 decreases bone and soft tissue metastatic burden by affecting tumor growth and tumorigenic potential^21^.

We have observed that ectopic expression of S18-2 in EC cell lines enhanced EMT^2^. Since S18-2 could immortalize cells with a de-differentiation phenotype and also induced stem cell markers, we evaluated the cooperation between S18-2 and CXCR4 in the progression of PCa and migration of PCa cells. We demonstrated that the degree of migration of PCa cells positively correlates with the expression level of S18-2, as the pattern of EMT markers does. We found that the *TWIST2* expression was induced upon S18-2 expression. Noteworthy, this effect was reversible - when S18-2 was downregulated, the *CXCR4* as well as *TWIST2* level was also reduced.

**Figure 10:**
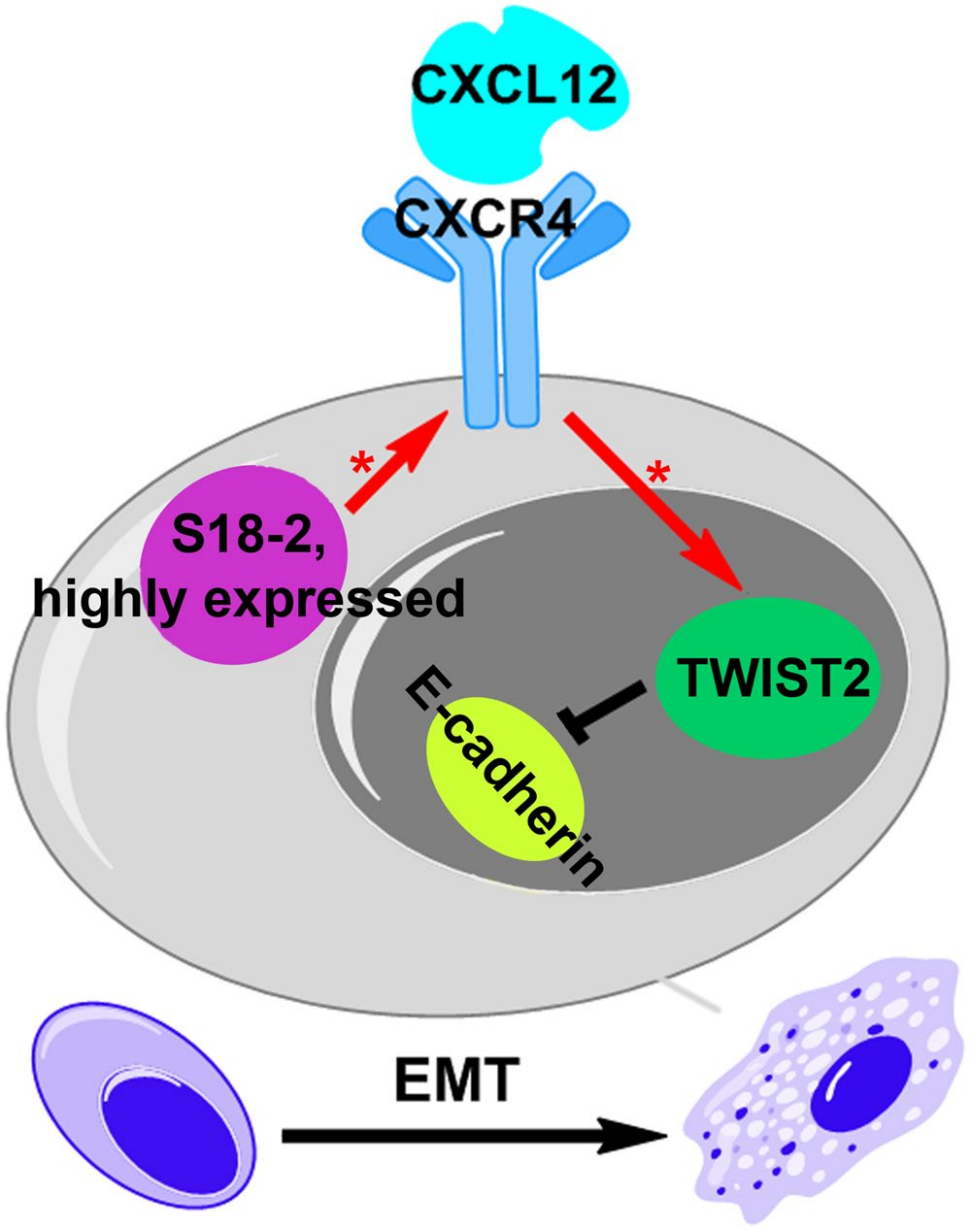
A putative mechanism of S18-2-dependent EMT induction. Our data show that overexpression of S18-2 in PC3 cells enriches the CXCL12-CXCR4 axis. This in turn results in upregulation of the expression of TWIST2, which is the repressor of E-cadherin, thus, EMT is enhanced. Therefore, the more cells express S18-2 protein, the better they gain the ability of migration as shown in zebrafish embryos. [*] indicates induction of gene.

It was shown earlier, that upon upregulation of CXCR4 the NF-κB pathway was induced in PC3 cells^22^. Importantly, via NF-κB pathway chemokines regulate expression of *TWIST1* and *TWIST2*^23^. Here we show that activation of CXCL12-CXCR4 axis upregulates the expression of *TWIST2*. Hence, we may speculate that overexpression of S18-2 leads to induction of CXCR4 expression and, consequently, *TWIST2* upregulation and repression of epithelial markers in PCa cells. As we show here, this results in EMT induction and the enhanced migration ability of cancerous cells (see Fig. 10).

The “go or grow” hypothesis of cancer proposes that the active migration suppresses cell proliferation^24,25^. We found that cells, overexpressing S18-2, migrate faster than the control cells. Noteworthy, they formed smaller tumors in SCID mice. Perhaps, this behavior could be explained by this hypothesis. Another possible reason of smaller tumor size of S18-2 overexpressing cells might be due to the fact that overexpression of S18-2 induce NK cells mediated cytotoxicity. We have shown such asymmetric behavior of S18-2 overexpressing rat embryonic fibroblast both, *in vivo* and *in vitro*^26^.

Concluding, we propose that the S18-2 protein through the enrichment of the CXCL12-CXCR4 axis increases the expression of the EMT specific transcription factor (*TWIST2*) that consequently enhances the migration of PCa cells. The current study may open a new insight and understanding of migration of PCa. Furthermore, analysis of S18-2 expression with other factors described here is under consideration in a larger cohort of clinical specimens, in order to identify prognostic biomarkers of metastatic advanced stage of PCa.

## Material and Methods

### Patient samples

Prostate tissue was obtained from 12 men diagnosed with localized PCa undergoing radical prostatectomy and 11 men diagnosed with bladder cancer undergoing cystoprostatectomy without findings of PCa at the subsequent pathological examination. All 11 patients without PCa were diagnosed with hyperplasia and different degree of inflammation (few had fibrosis also). Two experienced pathologists evaluated all the prostate specimens and assessed the Gleason grading in accordance with the 2016 WHO guidelines. All specimens were acquired under an Ethical Review Board in Uppsala-Örebro-approved protocol (2008/293) and written informed consent was obtained from each patient. All methods were performed in accordance with the guidelines and regulations as described in the protocol of ethical permission. Whole mount sections (4 µm) were used for immunohistochemical analysis. In this study we compared the group of hyperplasia with the PCa group (see supplementary Table S1).

### Immunohistochemistry

The S18-2 and CXCR4 protein expression was determined by immunohistochemistry of the paraffin-embedded tissue sections. Briefly, paraffin was dissolved in xylene and the tissues were rehydrated by stepwise washing with ethanol (EtOH) in phosphate-buffered saline (PBS) (96% and 70% EtOH). Tissues were then treated in a 2% solution of H_2_O_2_ in methanol at room temperature for 30 min to reduce background staining. Epitopes were exposed by hot citrate buffer (water bath, 96 °C for 15 min). Rabbit polyclonal anti-S18-2 (Proteintech Group, Inc., Chicago, IL, USA) 1:100 and mouse monoclonal anti-CXCR4 (MAB171-100, R&D Systems Abingdon, UK) 1:100 (10 mg/mL) antibodies were diluted in blocking buffer (2% bovine serum albumin, 0.2% Tween-20, 10% glycerol, and 0.05% NaN3 in PBS). Protein signals were visualized with the help of the EnVision™ Detection Peroxidase/DAB system (Dako, Glostrup, Denmark). Nuclei were stained with Mayer’s hematoxylin (Dako).

### Cell lines and transfections

The prostate cancer cell line PC3 and obtained sub-lines were cultured under normal conditions in IMDM medium supplied with 10% of bovine fetal serum and appropriate antibiotics at 37 °C. The PC3 cells were transfected with pBabe vector carrying the full-length cDNA of S18-2, using Lipofectamine 2000 (Life technologies, Carlsbad, CA, US) following the manufacturer protocol.

After transfection cells were selected, using medium supplemented with 5 µg/ml of puromycine. Then clones were tested for S18-2 expression in comparison with parental PC3. Two clones were chosen (PC3-S18-2-CL03 and PC3-S18-2-CL04) with different expression levels of S18-2.

### Immunofluorescence staining

Before immunostaining, cells were grown on coverslips. Cells were fixed in a mixture of cold methanol and acetone (1:1) at −20 °C. After rehydration in PBS, cells were stained with rabbit anti-MRPS18-2 (Proteintech, Manchester, UK) and pan-keratin (Dako). Hoechst 33258 (Sigma-Aldrich) was added to the secondary antibody (FITC conjugated swine anti rabbit) for DNA staining at a concentration of 0.1 mg/mL. The images were captured, using DAS microscope Leitz DM RB with a dual mode cooled charged coupled device (CCD) camera C4880 (Hamamatsu, Hamamatsu City, Japan).

### Western blotting

Whole cell lysates were prepared, by boiling the equal number of cells in Laemmli buffer at 95 °C for 10 m. Proteins were separated by sodium dodecyl sulphate-polyacrylamide gel electrophoresis (SDS-PAGE). After transfer, the nitrocellulose membranes were probed overnight at 4 °C, with primary antibodies. The following primary antibodies were used: Rabbit polyclonal anti-S18-2 (Proteintech Group, Inc., Chicago, IL, USA), E-cadherin (Cell Signaling Technology, Danvers, USA), cytokeratin 8 (Dako), β-catenin (Cell signaling Technology), actin and tubulin (Sigma-Aldrich). ECL kit, anti-mouse and anti-rabbit horseradish peroxidase-conjugated secondary antibodies, produced in sheep and donkey, respectively (GE Healthcare Bio-Sciences AB, Uppsala, Sweden), were used to visualize the protein bands.

### Zebrafish migration assay

Zebrafish 48 hpf embryos were used for this purpose. Before injection, cells were labelled with 1,1-Dioctadecyl-3,3,3,3-tetramethylindocarbocyanine perchlorate dye (DII) (Sigma Aldrich) at 10 μg/mL that enabled us to trace the cells in transparent embryos. The DII stained cells were injected into the perivitelline space of 48 hpf zebrafish embryos. Images were captured right after the injection to evaluate the number of cells injected to each embryo (Fig. 7A). The injected embryos were raised for three more days (before 120 hpf) and images were captured. Finally, the number of cells migrated to the tail of embryos were counted in each group. At least 20 embryos were injected with each cell line, and experiments were performed 3 times minimum for all cell types.

### CXCR4 blocking

The aggressive clone with high expression of S18-2 the PC3-S18-2-CL04 cells were stained with DII, trypsinized and incubated overnight with 2 µg/mL CXCR4 antibody or isotype control antibody (IgG2a, Becton Dickinson Biosciences, Germany). On next day cells were injected into zebrafish along with 100ng/mL CXCL12 and 10 µg/mL CXCR4 or control antibody.

PC3-S18-2-CL04 cells were transfected, using siRNA specific for S18-2 or control siRNA to analyze S18-2 effect on cell migration. After transfection cells were labeled with DII. Next day cells were injected to zebrafish embryos along with CXCL12 (100ng/µL). The number of cells migrated to tail of zebrafish embryos were counted after three days and images were captured.

### Animal Experiments using SCID Mice

The animal experiments were performed under ethical permission No.192/14, granted by Solna court in Stockholm, Sweden. All animal experiments were performed according to the guidelines and regulations as described in the ethical permission. Approximately 2 × 10^6^ cells were injected subcutaneously into SCID mice. To reduce the number of experimental animals but get statistically valid number of observations each mouse was injected into two locations and for each group of cells two mice were used (in total four injection points for each cell type). The tumor was palped every second day. When the size of tumor reached to maximum size allowed (1 cm^3^) according to the ethical permit the mice were sacrificed and tumors were excised.

### Flow Cytometry Analysis of CXCR4 Surface Expression

Approximately 5 × 10^5^ of PC3, PC3-S18-2-CL03 and PC3-S18-2-CL04 cells were used. After trypsinization cells were washed with PBS, incubated at 4 °C with PE conjugated anti CXCR4 antibody Clone 12G5 (Becton, Dickinson Biosciences Germany) or PE-Conjugated isotype control antibody (Becton, Dickinson Biosciences). Analysis was carried out with or without fixation of cells in 2% formaldehyde.

### CXCR4 activation

PC3 cells were cultured overnight in media without serum to starve them. Next dayf 100ng/ul of CXCL12 was added to media for CXCR4 activation. The cells were havested after 24 and 48 hours to extract RNA for *TWIST2* and *S18-2* mRNA expression analysis. The experiment was repeated at least three times.

### Quantitative PCR analysis

Total RNA was extracted from original PC3, PC3-S18-2-CL03 and PC3-S18-2-CL04. In parallel, the S18-2 was down regulated in PC3 cells using SMARTpool: ON-TARGETplus MRPS18B siRNA (Catalog No. L-013043-01-0005, Ge Healthcare), which is a pool of 4 siRNA. The efficiency of S18-2 specific siRNA is shown on Fig. 4E and Supplementary Figures S3E and S3F. Transfection of siRNA was performed using Dharmafect (Ge Healthcare). Cells were collected for extraction of RNA after 24 and 48 hours of transfection.

The 1 µg of each RNA sample was used to synthesize cDNA using Maxima First Strand cDNA Synthesis kit (Life technologies). For detection of gene expression Maxima SYBR Green/ROX qPCR (Life technologies) on 7500 PCR system (Applied Biosystems). The relative expression levels of the genes were calculated, using primers for *TATA binding protein (TBP) GAPDH* or *β-actin* as endogenous control. The following primers were used for amplification: *SNAIL* (NM_005985) - For_5′-CCTTCTCTAGGCCCTGGCT-3′, Rev_5′-AGGTTGGAGCGGTCAGC-3′; *TWIST2* (NM_057179) - For_5′-AGGCTCTCAGAAGAGGACCC-3′, Rev_5′-AAGGAAAAGAATAGCGGCGT-3′; *ZEB1* (NM_030751) - For_5′-CCAGGTGTAAGCGCAGAAA-3′, Rev_5′- TGCAGTTTGTCTTCATCATCTG-3′; *KLF8* (NM_007250) - For_5′-GACACTTCAGGAGTCCTCAGC-3′ Rev_5′-TGAATACCTGCATTGAGCCA-3′; *TBP* (NM_003194) - For_5′-GAGAGTTCTGGGATTGTACCG-3′ Rev_5′-ATCCTCATGATTACCGCAGC-3′ *GAPDH* (NM_001256799) – For_5′-ACCACCCTGTTGCTGTAGCCAA-3′ Rev_5′-GTCTCCTCTGACTTCAACAGCG-3′ *β-Actin* (NM_001101) - For 5′-CACCATTGGCAATGAGCGGTTC-3′ Rev_5′-AGGTCTTTGCGGATGTCCACGT-3′; *S18-2* (NM_014046) - For_5′-TCGTCGGAATAAAGTTGTTGG-3′ Rev_5′-GCAGACAAATTGCTCCAAGAG-3′.

Ct values were determined for the *TBP, β-actin or GAPDH* and for the other genes at the same threshold level in the exponential phase of the PCR curves. Relative quantification (comparative Ct (ΔΔCt) method) was used to calculate the relative expression levels. The q-PCR reactions were performed in triplicate at least three times and the standard deviation was calculated.

### Trans-well assay

Approximately 20000 cells were cultured in the upper chamber of each well of a Trans-well plate in IMDM media containing 2% FBS for 8 hours. When the cells attached to the membrane of trans-well plate, the media was replaced with the media with no serum, to starve the cells overnight. Next day, 100ng/ul CXCL12 was added only to the lower chamber of the trans-well plate and cells were incubated at 37 °C for 24 hours. Cells were washed with PBS twice and fixed in 4% formalin for 10 m at room temperature. Cells were stained with crystal violet. Cells on the upper chamber were removed using a cotton bud. Finally, images were captured for analysis. The experiment was repeated at least three times.

### Statistical analysis

GraphPad Prism software (version 7, GraphPad Software, La Jolla, CA, USA) was used for multiple comparisons of nonparametric criteria. Column analysis was performed using unpaired *t*-test. Two tailed *P*-values less than 0.05 were considered as statistically significant.

## Electronic supplementary material

Supplementary information
