# Supplementary material for: The MRPS18-2 protein levels correlate with prostate tumor progression and it induces CXCR4-dependent migration of cancer cells
Source: Sci Rep. 2018 Feb 2;8:2268. doi: 10.1038/s41598-018-20765-8 (PMC5797078; doi:10.1038/s41598-018-20765-8)
Supplement: Supplementary file 1 — Supplementary information [file 41598_2018_20765_MOESM1_ESM.pdf]

The MRPS18-2 protein levels correlate with prostate tumor progression and it induces CXCR4-dependent migration of cancer cells

Muhammad Mushtaq, Lasse Jensen, Sabina Davidsson, Oleksandr V Grygoruk, Ove Andrén3, Vladimir Kashuba, Elena Kashuba

Supplementary Information

Table S1. Description of prostate samples

Number	Diagnosis, based on HE staining	Gleason score	Additional information
1	Cancer	3+3	Inflammation
2	Cancer	3+3	A part of urethral gland is present
3	Cancer	3+4	
4	Cancer	4+4	Low differentiated cancer; small glands; cribriform glands. There are areas with hyperplasia and inflammation
5	Cancer	3+4	
6	Cancer	3+4	
7	Cancer	2+3	Stage I; highly differentiated cancer
8	Cancer	5+5	Stage IV; low differentiated cancer
9	Cancer	3+4	
10	Cancer	3+3	There is area with microcarcinoma (approximately 1 mm in diameter); inflammation
11	Cancer	3+3	There is the urethral part
12	Cancer	3+4	There are areas of sclerosis and the gland atrophy; a calcinated secret in atrophyc glands
13	Hyperplasia	NA*	
14	Hyperplasia	NA	A little of inflammation
15	Hyperplasia	NA	There are areas with the epithelium

			of urethra
16	Hyperplasia	NA	Combined glandular-fibrotic hyperplasia
17	Hyperplasia	NA	There is the gland atrophy
18	Hyperplasia	NA	There is area with microcarcinoma (approximately 1 mm in diameter)
19	Hyperplasia	NA	Inflammation; cancer in urethral part
20	Hyperplasia	NA	There are areas with the epithelium of urethra
21	Hyperplasia	NA	There is the gland atrophy and the epithelium of urethra
22	Hyperplasia	NA	There is areas with the epithelium of urethra
23	Stroma	NA	Cancer in urethral part

* NA – not applicable

Figure S1. mRNA expression level of S18-2 in prostate cancer

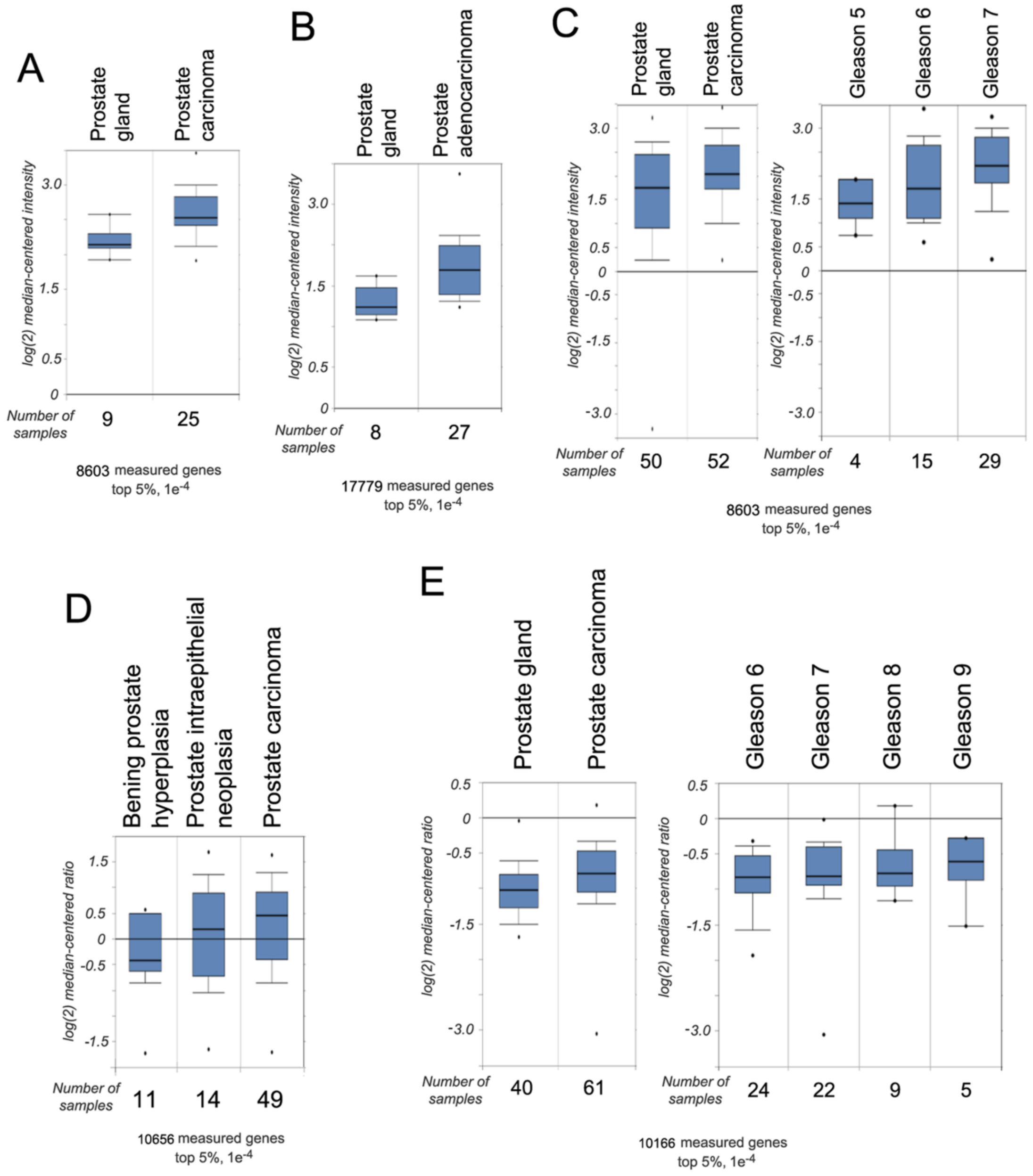

Figure S1. Expression analysis was performed with the help of Oncomine database. Expression of S18-2 is higher in PCa, compared with normal prostate gland (**A-E**). The data presented in (A) was described in [1]; (B) - in [2]; (C) - in [3]; (D) – in [4] and (E)- in [5].

Figure S2. Original gels of western blotting

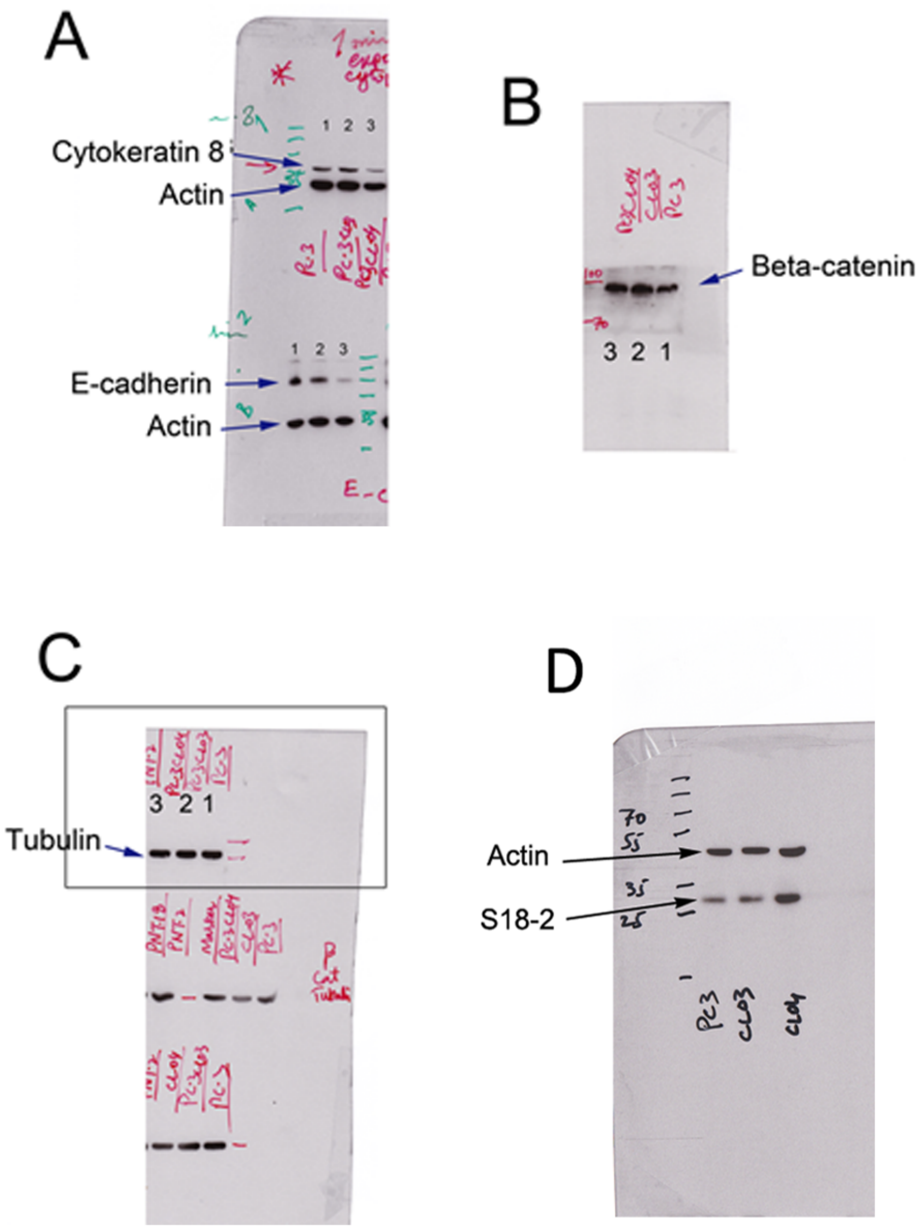

Figure S2. Original images of western blot analysis of EMT induction markers. Images were developed on a photofilm and scanned for presentation. In the Figure, lane (1) represents PC3, (2) - PC3-S18-2-CL03 and (3) - PC3-S18-2-CL04. **(A)** The 1st and 2nd rows of bands represent expression of cytokeratin 8 and actin (a loading control), respectively. The 3rd and 4th row of bands show the expression of E-cadherin and actin, respectively. **(B)** The expression levels of β-catenin. **(C)** The loading control for (B), tubulin is indicated within a square. **(D)** Expression level of S18-2 and its loading control actin.

Figure S3. Expression of genes, assessed by q-PCR

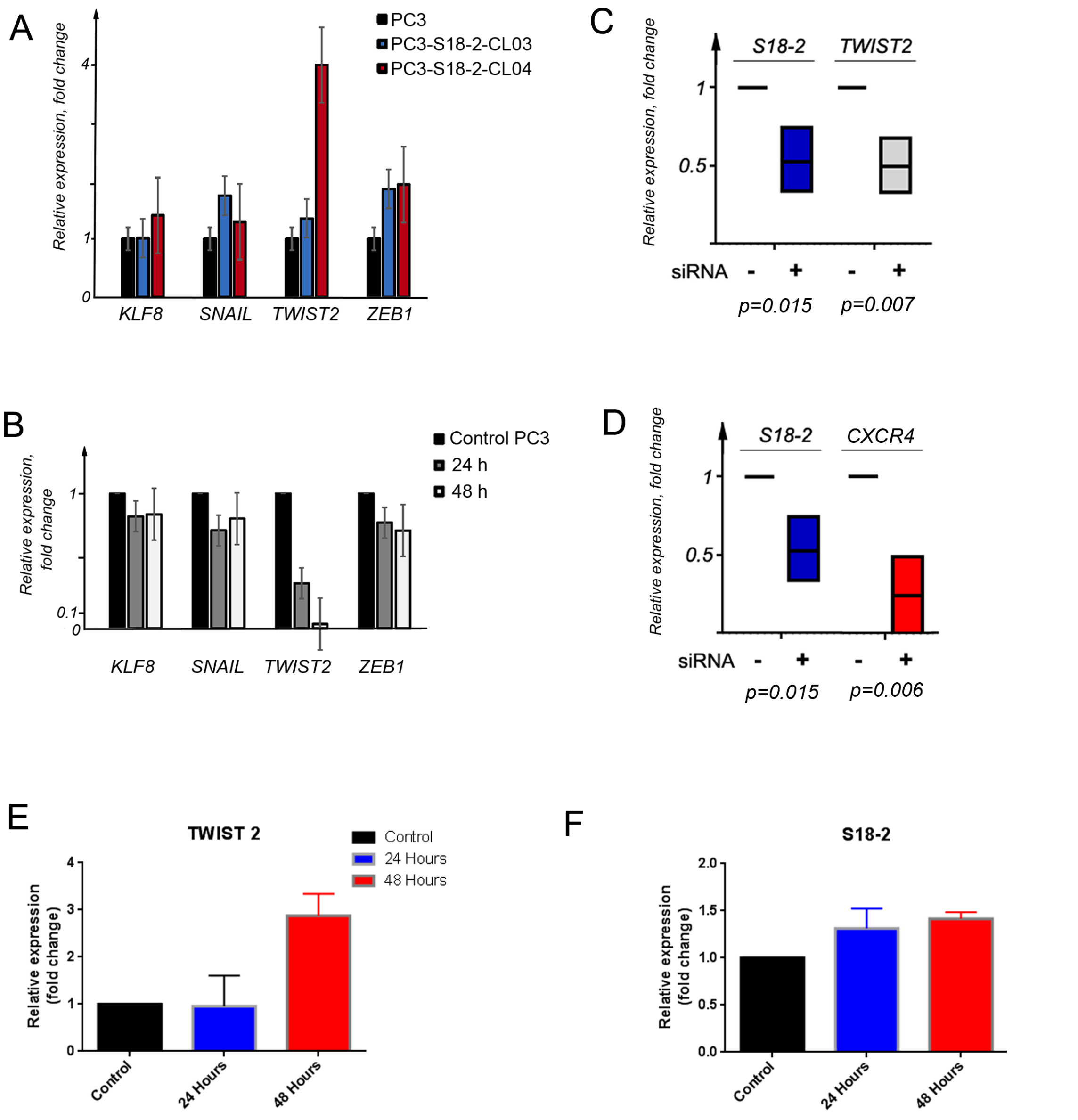

Figure S3. Expression of genes, assessed by q-PCR. For each gene, 3 reactions (each in triplicates) were run and a standard deviation was calculated. **(A)** The qPCR analysis of the indicated genes involved in EMT. q-PCR analysis showed that *TWIST2* was expressed at significantly higher levels in PC3-S18-2-CL04 than in the control cells. **(B)** The analysis of the expression pattern of the indicated transcription factors related to EMT at the mRNA level by q-PCR. The treatment during 24 h resulted in decreased *TWIST2* levels, and after 48 h *TWIST2* mRNA was below detection level. **(C)** The q-PCR analysis of *S18-2* and *TWIST2* expression. Three different siRNA transfections and three q-PCR reactions were performed

for each gene after 24 h. Medians of three q-PCR reactions were analyzed, using the GraphPad Prism software (version 7, GraphPad Software, La Jolla, CA, USA). The unpaired t test was applied to six values for each gene (controls - 3, siRNA – 3). (**D**) – as (C), but for the *CXCR4* gene. (**E**) The mRNA expression level of *TWIST2* after activation of CXCR4 by CXCL12 treatment. Cells were treated for 24 and 48 h. All the experiments were repeated at least three times. Medians of three q-PCR reactions were analyzed, using the GraphPad Prism software. (**F**) – as (E), but for *S18-2*.

References

1 Welsh, J. B. *et al.* Analysis of gene expression identifies candidate markers and pharmacological targets in prostate cancer. *Cancer research* **61**, 5974-5978 (2001).

2 Vanaja, D. K., Cheville, J. C., Iturria, S. J. & Young, C. Y. Transcriptional silencing of zinc finger protein 185 identified by expression profiling is associated with prostate cancer progression. *Cancer research* **63**, 3877-3882 (2003).

3 Singh, D. *et al.* Gene expression correlates of clinical prostate cancer behavior. *Cancer cell* **1**, 203-209 (2002).

4 Tomlins, S. A. *et al.* Integrative molecular concept modeling of prostate cancer progression. *Nature genetics* **39**, 41-51, doi:10.1038/ng1935 (2007).

5 Lapointe, J. *et al.* Gene expression profiling identifies clinically relevant subtypes of prostate cancer. *Proceedings of the National Academy of Sciences of the United States of America* **101**, 811-816, doi:10.1073/pnas.0304146101 (2004).
